# The Therapeutics of High Temperatures in Young Children

**Published:** 1886-07

**Authors:** 


THE THERAPEUTICS OF HIGH TEMPERATURES IN YOUNG CHILDREN.—By William Perry Watson, A. M., M. D., reprinted from *Archives of Pediatrics*. Publishers: John E. Potter & Co., Philadelphia. This is the substance of a clinical lecture delivered in the New York Polyclinic, and treats the subject in a very comprehensive and enlightened manner.

